# Manual prostate MRI segmentation by readers with different experience: a study of the learning progress

**DOI:** 10.1007/s00330-023-10515-4

**Published:** 2024-01-02

**Authors:** Fredrik Langkilde, Patrick Masaba, Lars Edenbrandt, Magnus Gren, Airin Halil, Mikael Hellström, Måns Larsson, Ameer Ali Naeem, Jonas Wallström, Stephan E. Maier, Fredrik Jäderling

**Affiliations:** 1 Department of Radiology, Sahlgrenska Academy, University of Gothenburg, Gothenburg, Sweden; 2 Department of Radiology, Sahlgrenska University Hospital, Gothenburg, Sweden; 3 Department of Molecular Medicine and Surgery (MMK), Karolinska Institutet, Stockholm, Sweden; 4 Department of Molecular and Clinical Medicine, Institute of Medicine, Sahlgrenska Academy, University of Gothenburg, Gothenburg, Sweden; 5 Department of Clinical Physiology, Sahlgrenska University Hospital, Gothenburg, Sweden; 6 Eigenvision AB, Malmö, Sweden; 7 Department of Radiology, Brigham and Women’s Hospital, Harvard Medical School, Boston, MA USA; 8 Department of Diagnostic Radiology, Capio S:T Göran’s Hospital, Stockholm, Sweden

**Keywords:** Magnetic resonance imaging, Prostate cancer, Learning curve, Artificial intelligence

## Abstract

**Objective:**

To evaluate the learning progress of less experienced readers in prostate MRI segmentation.

**Materials and methods:**

One hundred bi-parametric prostate MRI scans were retrospectively selected from the Göteborg Prostate Cancer Screening 2 Trial (single center). Nine readers with varying degrees of segmentation experience were involved: one expert radiologist, two experienced radiology residents, two inexperienced radiology residents, and four novices. The task was to segment the whole prostate gland. The expert’s segmentations were used as reference. For all other readers except three novices, the 100 MRI scans were divided into five rounds (cases 1–10, 11–25, 26–50, 51–76, 76–100). Three novices segmented only 50 cases (three rounds). After each round, a one-on-one feedback session between the expert and the reader was held, with feedback on systematic errors and potential improvements for the next round. Dice similarity coefficient (DSC) > 0.8 was considered accurate.

**Results:**

Using DSC > 0.8 as the threshold, the novices had a total of 194 accurate segmentations out of 250 (77.6%). The residents had a total of 397/400 (99.2%) accurate segmentations. In round 1, the novices had 19/40 (47.5%) accurate segmentations, in round 2 41/60 (68.3%), and in round 3 84/100 (84.0%) indicating learning progress.

**Conclusions:**

Radiology residents, regardless of prior experience, showed high segmentation accuracy. Novices showed larger interindividual variation and lower segmentation accuracy than radiology residents. To prepare datasets for artificial intelligence (AI) development, employing radiology residents seems safe and provides a good balance between cost-effectiveness and segmentation accuracy. Employing novices should only be considered on an individual basis.

**Clinical relevance statement:**

Employing radiology residents for prostate MRI segmentation seems safe and can potentially reduce the workload of expert radiologists. Employing novices should only be considered on an individual basis.

**Key Points:**

*• Using less experienced readers for prostate MRI segmentation is cost-effective but may reduce quality. *

*• Radiology residents provided high accuracy segmentations while novices showed large inter-reader variability. *

*• To prepare datasets for AI development, employing radiology residents seems safe and might provide a good balance between cost-effectiveness and segmentation accuracy while novices should only be employed on an individual basis. *

**Graphical abstract:**

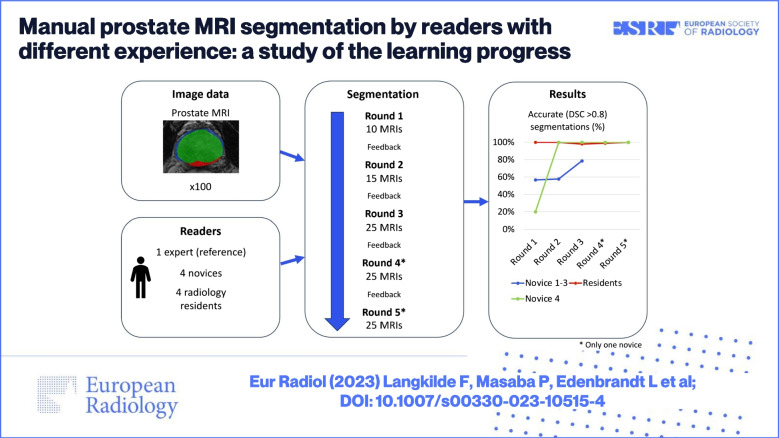

## Introduction

Magnetic resonance imaging (MRI) has become instrumental in the diagnostic pathway of prostate cancer (PCa) management [1, 2] and the number of MRI examinations in clinical practice is growing rapidly [3, 4]. In different scenarios, segmentation of the prostate gland is desirable, e.g., prior to fusion biopsies and prior to local radiotherapy [5–7]. Segmentation allows for prostate volume estimation which is a prerequisite when calculating prostate-specific antigen density [8]. Segmentation can be done manually and usually requires a radiologist’s expertise, but automated segmentation software is also available [9–11].

Manual segmentation is time-consuming and prone to inter-reader variability even among experts [12–14]. Artificial intelligence (AI) has shown promise in improving the speed of manual prostate MRI segmentation [15, 16]*.* However, large amounts of high-quality training data are needed [17] to obtain reliable AI algorithms for automated segmentation. Expert radiologists are the most qualified to generate such high-quality training data, but in view of the necessary extensive time commitment, engagement of less experienced readers may be a worthwhile consideration but may reduce overall data quality [18].

Although several studies have examined inter-reader variability for prostate segmentation, to the best of our knowledge, no study has investigated the learning rate with continuous feedback for readers with different levels of experience [13, 14]. This study aims to map the learning progress of less experienced readers in prostate MRI segmentation.

## Material and methods

### Ethical considerations

The present study was approved by the Swedish Ethical Review Authority under the registration number 2019–04453.

### Image data

One hundred bi-parametric prostate MRI scans were retrospectively selected from the ongoing Göteborg Prostate Cancer Screening 2 Trial (G2 trial). This study population consists of approximately 40,000 men aged 50–60 years, recruited from Gothenburg and six surrounding municipalities. The G2 trial is described in detail elsewhere [19].Written informed consent was obtained from all subjects before inclusion in the G2 trial. The MRI scans used in the present study were randomly selected for study inclusion between the dates of January 1, 2017, and March 22, 2019. The scan protocol included T_2_-weighted (T2W) imaging in three orthogonal planes and diffusion-weighted imaging (DWI) in the axial plane according to the PI-RADS v2.1 guidelines [20]. The axial T2W images, which were used for manual segmentation, were obtained with a field of view of 180 × 180 mm^2^, an acquisition matrix of 200 × 212, and a slice thickness of 1.5 mm. All examinations were performed using the same Philips Achieva dStream 3-T MRI scanner. The expert radiologist determined if the image quality was adequate or if the case should be excluded.

### Readers

Nine readers with varying degrees of segmentation experience were involved: one expert radiologist (“expert,” 13 years of experience of prostate MRI and more than 1000 prostate MRI segmentations), two experienced radiology residents (“experienced residents,” both had 3 years of prostate MRI experience and 100–200 prostate MRI segmentations), two inexperienced radiology residents (“inexperienced residents,” no prostate MRI experience), and four inexperienced medical students (“novices,” no medical image segmentation experience). The experienced residents were both in their 3rd year of residency and the inexperienced residents were in their 1st respectively 5th year of residency while the novices were enrolled in the 5th year of medical school.

The radiologists performed the segmentation during working hours. One of the novices (novice 4) participated as a part of his master thesis during his last year of medical school.

### Segmentation procedure

**Fig. 1:**
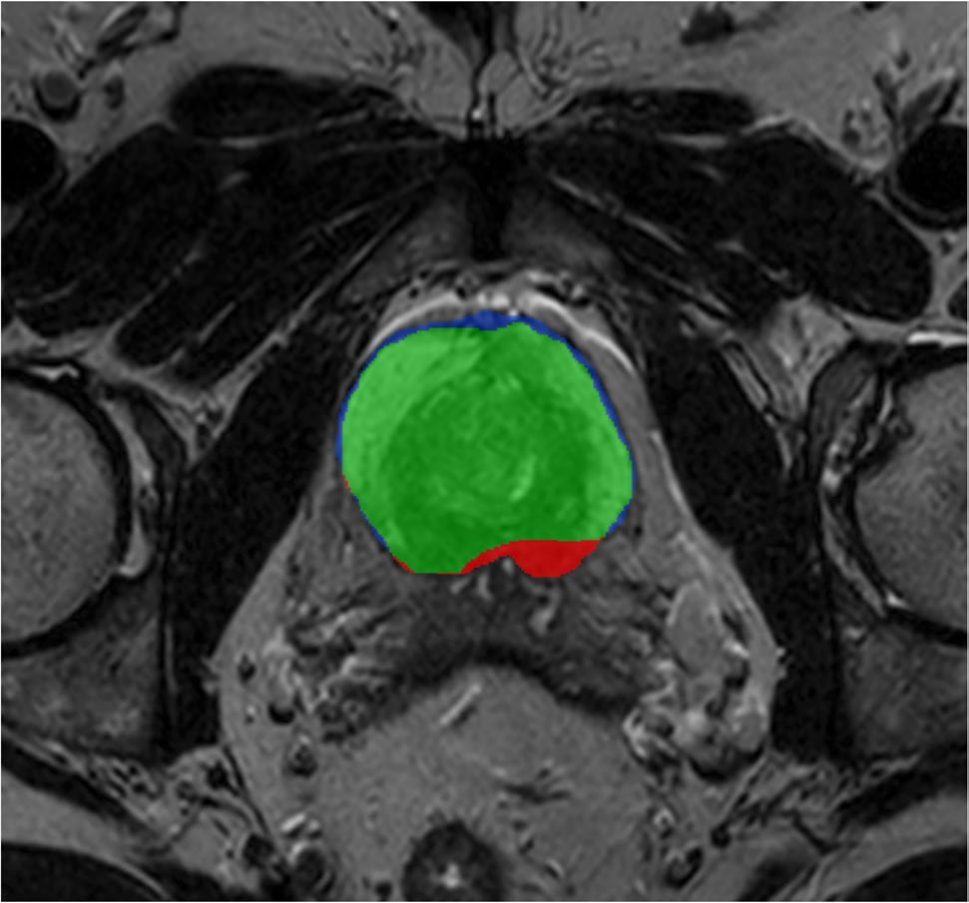
T2-weighted axial image from the feedback session in RECOMIA where green color shows segmented areas in agreement, blue color areas segmented by the study participant only, and red color areas segmented by the expert only

All MRI images were anonymized, and no clinical or technical image information was provided to the readers. The task was to segment the whole prostate gland excluding the seminal vesicles. All acquired MRI series were available to the reader, but manual slice-by-slice segmentation was performed on the axial T2W series only, which consisted of between 44 and 60 slices. The segmentation software RECOMIA was used [21]. Readers received prior oral and written instructions on using the software. Only one reader (experienced resident 1) had used the software before, segmenting approximately 100 prostate cases. None of the readers had received training or feedback on prostate segmentation from the expert prior to the study. None of the inexperienced residents or novices had attended lectures, courses, or similar theoretical training prior to the study. Readers were free to use resources available elsewhere. The expert segmented all 100 MRI scans, and these segmentations were designated as references. The 100 MRI scans were divided into five segmentation rounds (see Table 1). All cases were segmented in the same order by all readers. Five readers segmented all cases while three readers (novices 1–3) only segmented the first 50 cases (rounds 1–3). After each round, a 30-min one-on-one feedback session between the expert and the reader was held online, using computer screen sharing. All feedback sessions were held in Swedish and all readers were native Swedish speakers. Before each feedback session, the expert was provided with his own segmentations (“reference”) and the reader’s segmentations, with non-overlapping segments highlighted in the software RECOMIA (see Fig. 1). The expert selected between three and five cases, for which he provided visual and oral feedback on the reader’s annotations and pointed out systematic errors and potential improvements for the next round. The next round started immediately after the feedback session given for the prior round. Each reader recorded the time spent on each case.

**Table 1:** Segmentation rounds

Round	Cases	Days available to readers
1	1–10	8
2	11–25	19
3	26–50	8
4	51–75	15
5	76–100	13
All	1–100	63

The cases included and the number of days available to the readers for each round

### Evaluation and statistical analysis

Four different evaluation metrics were used: Dice similarity coefficient (DSC), Hausdorff distance (HD), average surface distance (ASD), and volume difference (VD). DSC is a similarity metric measuring the proportion of overlapping pixels between a pair of image segmentations. DSC is the most widely used evaluation metric and comparison of figures and tables focuses therefore on DSC. For the purpose of the study, we considered segmentations with a DSC of above 0.8 to be accurate and all others inaccurate regardless of the other metrics, as used in other publications [13, 14]. The HD and ASD are dissimilarity metrics describing the longest distance and the average distance between the margins of the segmentations, respectively [22]. The VD between segmentations is expressed as a percentage compared to the expert. The prostate was also divided into three parts with an equal number of slices in the longitudinal direction according to the expert’s segmentation and accuracy was measured in the three parts called base, mid-gland, and apex.

A comparison of mean DSC between rounds 1 and 3 was performed for all individual readers separately with an independent *t*-test using SPSS (version 29.0), in order to evaluate if any change in DSC had occurred. A *p*-value below 0.05 was considered significant.

No additional statistical analyses were carried out due to the limited number of readers.

## Results

The median patient age was 57 years [range 50–63 years], the median patient prostate-specific antigen level was 3.3 ng/mL [range 1.8–29 ng/mL], and the median MRI prostate gland volume determined by the expert reader was 39 cm^3^ [range 16–198 cm^3^]. One case was excluded due to excessive artifacts from bilateral hip prostheses. This case was replaced with another randomly selected case.

**Fig. 2:**
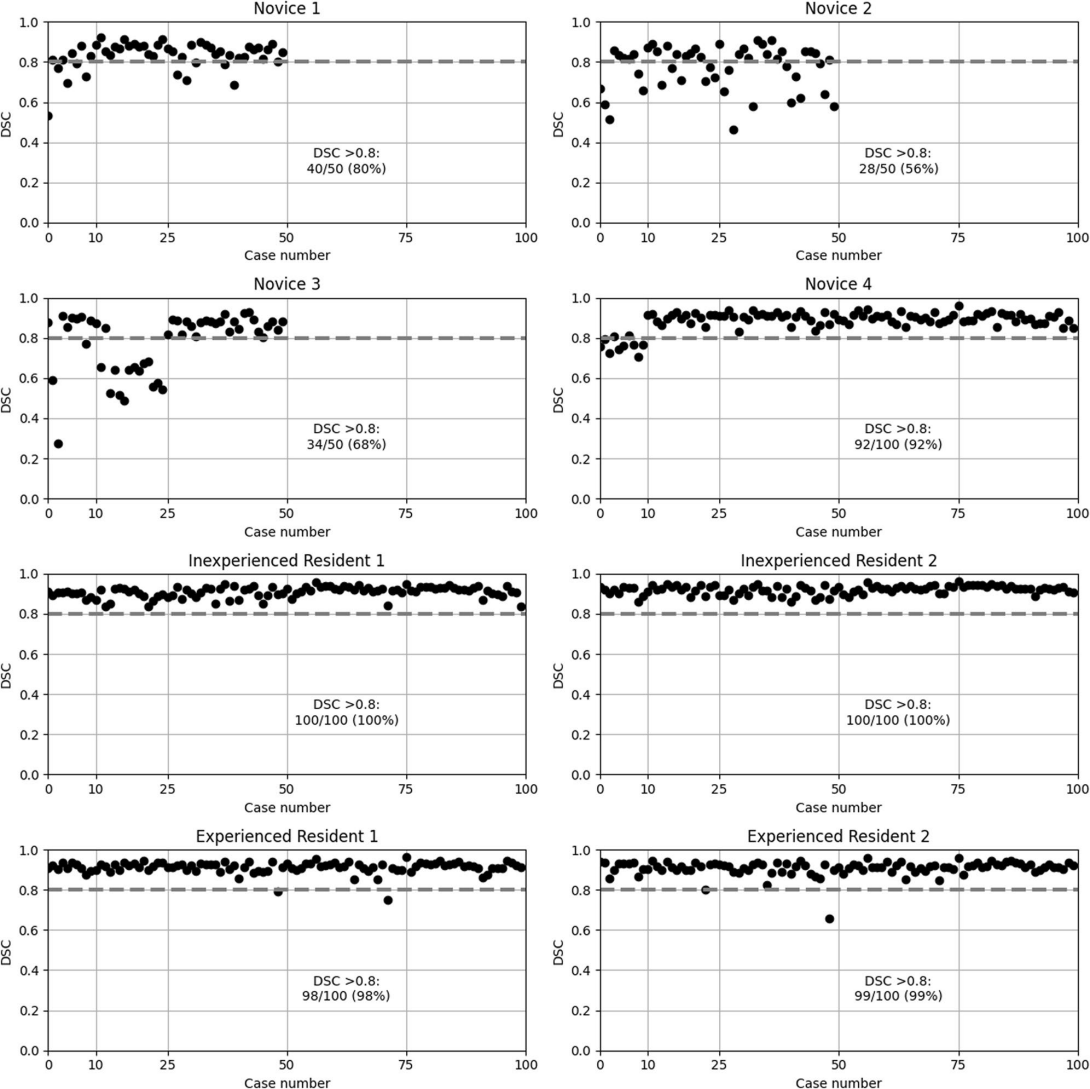
Dice similarity coefficient (DSC) for all readers and all cases with the expert’s segmentations as reference. Feedback sessions were held after cases 10, 25, 50, and 75. DSC above 0.8 was considered accurate (dashed line), the number and percentage of accurate segmentations for each reader are reported. There is high inter-reader variability among novices. Novice 4 showed a clear improvement after round 1 and had only accurate segmentations in subsequent rounds. Novice 3 showed low values in round 2 but then improved to round 3. Novices 1 and 2 had no clear improvement but novice 1 had an overall higher DSC. All residents had consistently high DSC with only three cases of inaccurate segmentations (case 49 for both experienced residents and case 72 for experienced resident 1)

**Fig. 3:**
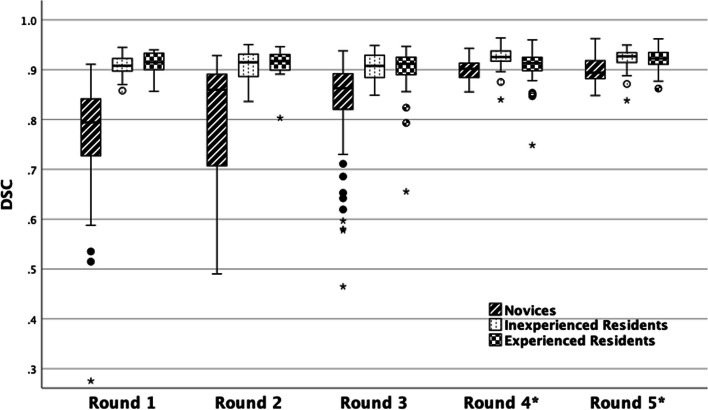
Boxplot of whole gland segmentation accuracy, with the expert’s segmentations as reference, measured by Dice similarity coefficient (DSC) for readers with different levels of experience and for all rounds. Novices, inexperienced residents, and experienced residents are grouped separately. Novices 1–3 participated in rounds 1–3 (cases 1–50), and all others participated in rounds 1–5 (cases 1–100). Each box covers the data spectrum for a segmentation round and is marked by median, lower, and upper quartiles. Outliers and extreme values are marked with a circle and star respectively and are set according to Tukey’s method. *Only one novice

Figure 2 shows DSC for all readers and all cases and Fig. 3 summarizes DSC for all readers and all cases. From the plots in Fig. 2, it can be appreciated that there was a larger variation among novices than among residents. All reader groups had a median DSC of above 0.8 except the novices in round 1. The novices had a total of 194 accurate (DSC > 0.8) segmentations out of 250 (77.6%). The inexperienced residents had a total of 200/200 (100%) accurate segmentations and the experienced residents 197/200 (98%) accurate segmentations. In round 1, the novices had 19/40 (47.5%) accurate segmentations, in round 2 41/60 (68.3%), and in round 3 84/100 (84.0%) indicating learning progress which also can be appreciated in the figures. Novice 4 had 2/10 (20.0%) accurate segmentations in round 1 and 90/90 (100.0%) in rounds 2 through 5.

Table 2 summarizes all readers’ results over all rounds for all four evaluation metrics. When comparing the mean DSC for all readers separately for rounds 1 and 3, only novice 4 showed a statistically significant improvement (*p* < 0.001; see Table 3). The average time spent on each case for each reader and round is presented in Table 3.

**Table 2:** Whole gland segmentation accuracy

		Round 1		Round 2		Round 3		Round 4		Round 5		All rounds	
		Median	IQR	Median	IQR	Median	IQR	Median	IQR	Median	IQR	Median	IQR
DSC	Novices	0.79	0.11	0.86	0.18	0.86	0.07	*	*	*	*	0.87	0.11
	Inexperienced residents	0.91	0.02	0.92	0.04	0.90	0.04	0.92	0.02	0.92	0.02	0.92	0.03
	Experienced residents	0.91	0.03	0.92	0.03	0.91	0.03	0.91	0.03	0.92	0.02	0.92	0.03
ASD [mm]	Novices	2.16	1.56	1.25	2.02	1.20	0.55	**	**	**	**	1.19	1.39
	Inexperienced residents	0.90	0.25	0.70	0.51	0.77	0.33	0.58	0.15	0.56	0.23	0.65	0.32
	Experienced residents	0.78	0.29	0.72	0.31	0.73	0.38	0.68	0.23	0.60	0.16	0.68	0.26
HD [mm]	Novices	15.84	7.14	11.23	5.29	9.98	3.73	***	***	***	***	9.78	5.24
	Inexperienced residents	7.97	1.50	6.32	2.80	6.83	3.66	5.99	1.38	5.98	2.18	6.32	2.39
	Experienced residents	7.34	3.82	8.26	3.38	7.19	5.34	7.32	2.62	6.03	2.32	7.09	3.67
VD [%]	Novices	− 7.07	28.85	− 4.55	25.20	− 3.90	25.93	****	****	****	****	− 6.51	26.48
	Inexperienced residents	1.40	10.79	− 0.72	11.65	4.94	11.90	2.79	9.59	− 3.24	7.67	2.20	10.69
	Experienced residents	− 5.97	14.90	3.26	8.70	− 5.99	9.44	− 6.80	6.18	− 3.12	13.17	− 4.59	11.02

Whole gland segmentation accuracy, with the expert’s segmentations as reference, over all rounds as measured by four evaluation metrics: Dice similarity coefficient (DSC), average surface distance (ASD), Hausdorff distance (HD), and volume difference (VD). *IQR* interquartile range

^*^Median DSC solely for novice 4, round 4: 0.9 (IQR 0.03) and round 5: 0.89 (IQR 0.04)

^**^Median ASD solely for novice 4, round 4: 0.82 (IQR 0.23) and round 5: 0.78 (IQR 0.27)

^***^Median HD solely for novice 4, round 4: 6.68 (IQR 1.63) and round 5: 6.07 (IQR 1.03)

^****^Median VD solely for novice 4, round 4: − 8.27 (IQR 6.48) and round 5: − 14.06 (IQR 8.07)

**Table 3:** Data per reader with average time per case and change in mean DSC in round 1 vs 3

Reader	Average time per case (min:ss)						Mean DSC change, round 1 vs 3 (confidence interval)	*p*-value
	Round							
	1	2	3	4	5	All		
Novice 1	20:30	18:57	19:04			19:30	0.06	0.088
							(− 0.01, 0.13)	
Novice 2	08:00	08:00	06:48			07:36	0.03	0.464
							(− 0.06, 0.13)	
Novice 3	18:00	12:23	17:16			15:53	0.08	0.248
							(− 0.07, 0.23)	
Novice 4	18:51	28:34	31:25	28:57	20:59	25:45	0.14	< 0.001
							(0.11, 0.16)	
Inexperienced resident 1	38:48	29:08	34:58	32:34	28:05	32:43	0	0.55
							(− 0.01, 0.02)	
Inexperienced resident 2	28:48	12:56	08:41	08:26	07:44	13:19	− 0.01	0.576
							(− 0.03, 0.01)	
Experienced resident 1	11:32	10:04	07:02	05:48	04:44	07:50	− 0.01	0.524
							(− 0.02, 0.01)	
Experienced resident 2	10:10	09:13	06:36	05:41	05:08	07:22	− 0.02	0.232
							(− 0.05, 0.01)	

Average segmentation time per case for each reader and round is reported. An independent *t*-test was performed to test for change in DSC between rounds 1 and 3. Mean DSC change for each reader between round 1 and round 3 and *p*-values are reported (*p* < 0.05 was considered significant). Only novice 4 had a significant increase in DSC from round 1 to round 3 (*p* < 0.001)

**Fig. 4:**
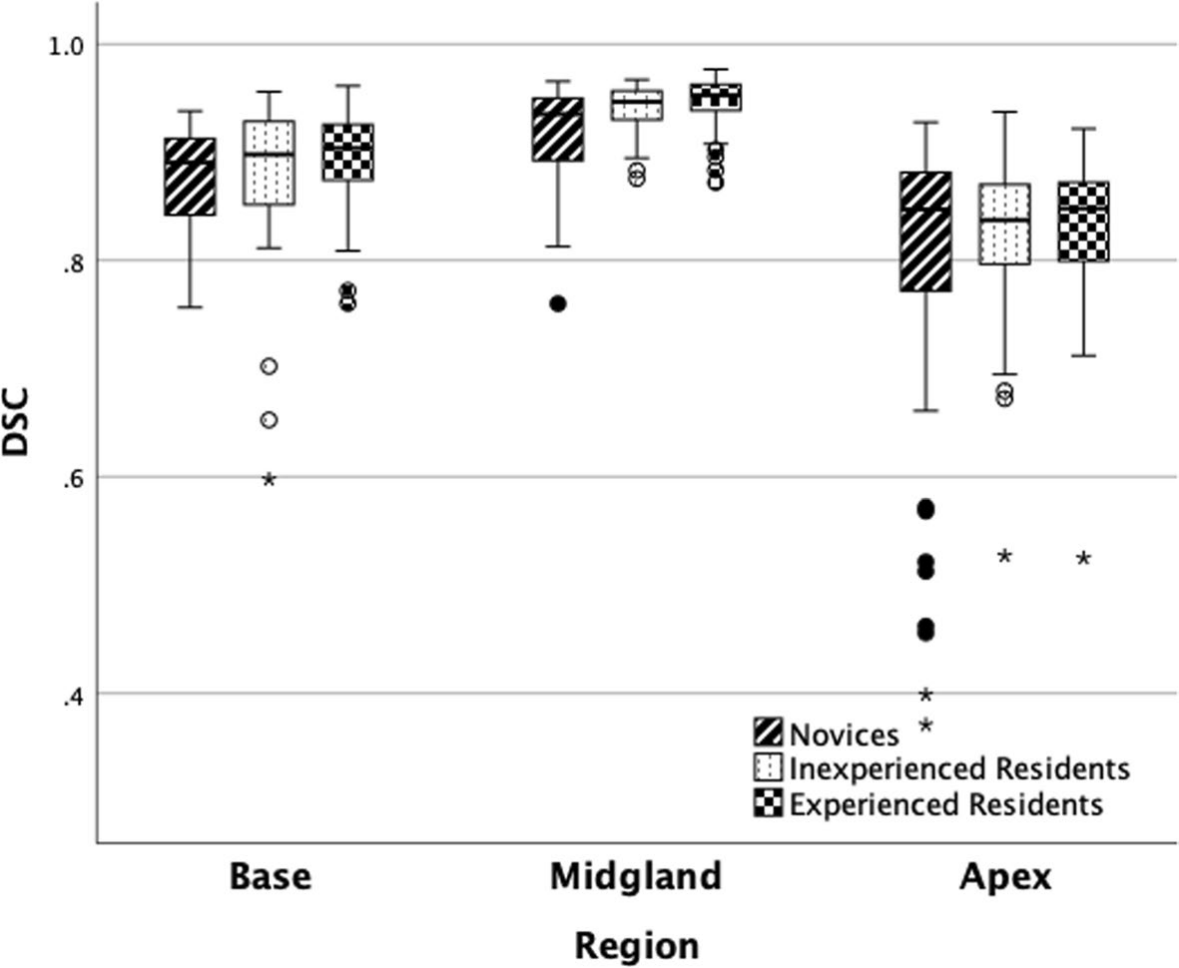
Boxplot of segmentation accuracy measured by Dice similarity coefficient per region of the prostate. Novices, inexperienced residents, and experienced residents are grouped separately. Novices 1–3 participated in rounds 1–3 (cases 1–50), and all others participated in rounds 1–5 (cases 1–100). Each box covers the data spectrum and is marked by median, lower, and upper quartiles. Outliers and extreme values are marked with a circle and star respectively and are set according to Tukey’s method

DSC was highest in the mid-gland and lower in the apex and base as shown in Fig. 4.

## Discussion

We studied the learning progress of prostate MRI segmentation for readers with varying levels of experience. Our results show that the radiology residents in this study, regardless of prior experience, had a DSC of above 0.8 in all segmentations except three, i.e., a high similarity with the expert. The major benefit of these results is that it seems safe to employ radiology residents to create data labels that can be used as training data for automatic prostate segmentation models. Among novices, there was a large variation, with novice 4 standing out with no segmentation below 0.8 in rounds 2 through 5, probably due to a high degree of motivation. This suggests that employing novices should only be considered on an individual basis.

To our knowledge, only two earlier studies have thoroughly investigated aspects of the performance of manual segmentation of the prostate gland. Becker et al investigated the inter-reader variability of six readers in the different prostatic zones while Montagne et al examined the impact of prostate morphology and inter-reader variability of prostate segmentation [13, 14]. Montagne et al observed a significant association between increasing expertise and reduced inter-reader variability. In whole gland prostate segmentation, Montagne et al reported a mean inter-reader DSC of 0.92 and Becker et al 0.86 for radiologists, results which are well in line with our results of a median DSC of 0.92 for radiology residents. This suggests that the residents in our study attain accuracy at the same level as radiologists in the two prior studies. Compared with automated approaches, Jimenez-Pastor et al achieved a DSC of 0.88 for whole gland segmentation, which is similar to the residents in the present study [23].

The average time spent on each case varied a lot between readers, ranging from approximately 7 to 33 min. Variations in segmentation times could be explained by several factors such as experience, motivation, and carefulness but these were not further analyzed in our study.

The median DSC for all readers was lower in the apex and the base and higher in the mid-gland, which is in line with the results of both Becker et al and Montagne et al [13, 14]. One reason behind the difficulties in the apex and base may be the higher risk of partial volume effects in these areas since axial images were used for segmentation. A potential way of increasing the segmentation accuracy in the apex and base could be to also use sagittal and coronal images for segmentation, although this is more time-consuming.

When evaluating segmentation accuracy, the objective is to identify segmentation errors that are clinically relevant, i.e., errors that are consequential for the diagnosis or the treatment of the patient. A single quantitative metric that reflects all relevant information for comparing segmentations would be preferable, but different metrics tend to convey slightly different segmentation errors. Therefore, four different metrics were used but the focus was put on DSC. Determining the level of each metric that corresponds to a clinically adequate or acceptable segmentation is another problem. We considered DSC above 0.8 as “accurate,” as used in other publications [13, 14]. The three additional metrics showed similar trends as DSC, supporting the choice of DSC as the primary outcome.

A strength of our study was that all examinations were obtained from a single PCa screening population (the G2 trial) and all MRI scans were acquired using the same scanner and protocol. When studying the learning progress of prostate gland segmentation, this can be considered a strength since it reduces the risk of other factors spuriously affecting the results. However, it might limit generalization and our findings may not necessarily translate to images acquired on other scanners or other scanning protocols. Segmentations were performed under the same circumstances and all readers received feedback from the same expert in an equivalent manner, which is another strength of the study.

A limitation of the present study is that three of the four novices only segmented the first 50 cases. Nevertheless, we believe that the data on 50 cases provides important information on the limited progression rate of novices in segmenting the prostate.

Including additional experts in our study could have provided a better reference to evaluate and compare the non-experts. However, in the present study, only one expert was available, which is a potential weakness of the study.

We had a high number of cases for the readers to segment, but the limited number of readers may be a limitation of the study.

In the near future, we will have to rely on accurate manual segmentations for the training of deep neural networks, until we have algorithms that serve us with such. In the making of those, training and motivation will be the steppingstone in that endeavor.

Radiology residents, regardless of prior experience, showed high segmentation accuracy.

Novices showed larger interindividual variation and lower segmentation accuracy than radiology residents. To prepare datasets for AI development, employing radiology residents seems safe, after an initial introduction and evaluation, and continuous feedback might provide a good balance between cost-effectiveness and segmentation accuracy. Employing novices should only be considered on an individual basis but with some caution due to higher inter-reader variability compared to more experienced readers.

## Abbreviations

AI: Artificial intelligence
ASD: Average surface distance
DSC: Dice similarity coefficient
DWI: Diffusion-weighted imaging
HD: Hausdorff distance
PCa: Prostate cancer
T2W: T_2_-weighted imaging
VD: Volume difference

## Declarations

### Guarantor

The scientific guarantor of this publication is Fredrik Jäderling.

### Statistics and biometry

Marianne Månsson, PhD, Associate Professor, Statistician, Department of Urology, Institute of Clinical Sciences, Sahlgrenska Academy at the University of Gothenburg, kindly provided statistical advice for this manuscript.

### Study subjects or cohorts overlap

Some study subjects or cohorts have been previously reported in:

Hugosson J, Månsson M, Wallström J, Axcrona U, Carlsson SV, Egevad L, et al Prostate cancer screening with PSA and MRI followed by targeted biopsy only. New England Journal of Medicine. 2022;387(23):2126–37.

### Methodology

• retrospective

• observational

• performed at one institution
